# Mitigation of metabolic dyshomeostasis by glucocorticoid-receptor antagonism: Insights from animal and human studies

**DOI:** 10.17179/excli2020-2814

**Published:** 2020-09-09

**Authors:** Aishwariya Madhavan, Kusuma Murali, Vaishnavi Raghavendra, Apurva Kumar Ramesh Joshi

**Affiliations:** 1 Department of Biochemistry, School of Sciences, Jain (Deemed to be University), Jayanagar 3rd Block, Bangalore, Karnataka, India 560041

## ⁯

***Dear Editor,***

Glucocorticoid hormones are steroidal signaling molecules produced by cortex of the adrenal gland. While acute glucocorticoid response is critical for immunomodulation and metabolic homeostasis, chronic elevated glucocorticoid levels have been recognized as a risk factor for metabolic syndrome (Wang, 2005[55]). Much of the understanding of consequences of excess glucocorticoids on metabolic homeostasis has come from observations on pathology associated with Cushing's syndrome. While incidence of Cushing syndrome is extremely low (Lindholm et al., 2001[35]), chronic exposure to excess glucocorticoids is a more realistic issue and needs to be taken into consideration. It is now well recognized that abnormalities such as diabetes/impaired glucose tolerance, obesity, hypertension and dyslipidemia are highly prevalent among patients of Cushing's syndrome (Chanson and Salenave, 2010[9]). A cross sectional study involving Cushing's syndrome patients clearly establishes the correlation between endogenous hypercortisolemia and metabolic abnormalities. The said study demonstrates that prompt diabetes was evident in 38 % of patients and fasting blood glucose, oral glucose tolerance test area under the curve (AUC) and HbA1C levels positively correlating with urinary free cortisol (Friedman et al., 1996[19]). The effect of glucocorticoids on metabolic homeostasis can also be discerned by the analysis of effects of corticosteroid therapy on glycemic regulations. Synthetic corticosteroids are the choice of drugs to treat various health issues such as asthma, chronic pulmonary obstructive disorders and rheumatoid arthritis. Panthakalam et al. (2004[40]) reported that 9 of 102 patients receiving glucocorticoid therapy for rheumatoid arthritis developed diabetes while pre-existing state of diabetes in another 6 worsened during the course of treatment (Panthakalam et al., 2004[40]). A retrospective analysis of medical data of patients who were hospitalized at general service of a hospital revealed that 64 % of patients receiving exogenous corticosteroid for at least 2 days developed hyperglycemia. This study demonstrates high prevalence of hyperglycemia among those receiving corticosteroid therapy and indicates that people with a history of diabetes before corticosteroid treatment are likely to develop hyperglycemia on corticosteroid therapy or due to other multiple co-morbidities (Donihi et al., 2006[13]).

Indeed, many experimental and clinical studies lend strong support to the view that excess glucocorticoid levels share causal relationship with various components of metabolic syndrome. Brunner et al. studied changes in autonomic cardiac activity and neuroendocrine functions in metabolic syndrome patients (n=30 vs. 153 control) of Whitehall II cohort. Interestingly, they observed that excretion (24 h) of a cortisol metabolite and normetanephrine increased in patients, in addition to higher levels of circulating interleukin-6 and C-reactive peptide (Brunner et al., 2002[7]). Analysis of cross-sectional data from the Paris Prospective Study revealed strong association of high systolic blood pressure with cortisol, blood glucose, heart rate, and free fatty acids (Filipovský et al., 1996[16]). A study conducted with young overweight Latino subjects revealed increased cortisol and fasting insulin levels in addition to increased 2 h glucose and insulin (during OGTT) levels among youth with metabolic syndrome. Further, systolic and diastolic blood pressure, fasting glucose levels and intra-abdominal fat tissue mass in subjects with MS were reported to correlate with cortisol levels, indicating that excess cortisol may have far reaching consequences on metabolic homeostasis (Weigensberg et al., 2008[58]). Similarly, another study conducted in obese children and adolescents revealed that circulating ACTH and cortisol levels were higher in metabolic syndrome subjects, who also had higher fasting glucose and insulin, increased systolic and diastolic blood pressure, and increased triglyceride levels (Sen et al., 2008[50]). To summarize, many human-subject based studies demonstrate that the excess glucocorticoid level is associated with many defining components of metabolic syndrome viz., increased waist circumference (Pasquali and Vicennati 2000[41]), increased triglyceride levels (Friedman et al., 1996[19]; Phillips et al., 1998[43]; Ward et al., 2003[56]), hypertension, increased blood glucose (Brunner et al., 2002[7]; Weigensberg et al., 2008[58]; Sen et al., 2008[50]) and insulin resistance (Phillips et al., 1998[43]; Ward et al., 2003[56]; Reinehr and Andler, 2004[47]).

Human subject-based studies clearly establish the association between cortisol and various metabolic aberrations associated with metabolic syndrome. While they offer clear perspectives on these correlations, much of the understanding of mechanisms responsible for the diabetogenic effects of glucocorticoids come from preclinical studies involving *in vitro* systems and experimental animal models. Owing to the fact that GCs are associated with insulin resistance, many authors have investigated the direct effect of GCs on beta cell functions employing isolated islets or insulin producing cell lines. Direct exposure to GCs appears to inhibit insulin release *in vitro *(Barseghian and Levine, 1980[2]; Gremlich et al., 1997[23]; Lambillotte et al., 1997[31]; Jeong et al., 2001[29]; Shinozuka et al., 2001[51]; Ullrich et al., 2005[54]; Zawalich et al., 2006[62]), an outcome which appears to be mediated by post-translational degradation of Glut2 protein (Gremlich et al., 1997[23]). Interestingly, this inhibitory effect is abolished by mifepristone, a glucocorticoid-antagonist, indicating involvement of receptor mediated mechanisms (Lambillotte et al., 1997[31]; Zawalich et al., 2006[62]). Despite compelling *in vitro* evidences for antagonistic effects of GCs on various aspects of beta cell functioning, such results were difficult to reproduce *in vivo*. On the contrary, paradoxically enough, experimental (Haber and Weinstein, 1992[24]; Giorgino et al., 1993[22]; Weinstein et al., 1993[59]; Holland et al., 2007[27]; Rafacho et al., 2009[46]; Protzek et al., 2014[45]) and human studies (Beard et al., 1984[3]; Willi et al., 2002[61]; Nicod et al., 2003[36]; Binnert et al., 2004[6]) demonstrate that administration of glucocorticoids results in hyperinsulinemia as a result of augmented beta cell function to compensate for peripheral insulin resistance. 

Glucocorticoids are known for transcriptional activation genes of gluconeogenesis enzymes like G6Pase (Argaud et al., 1996[1]), phosphoenolpyruvate carboxykinase (PEPCK) (O'Brien et al., 1990[38]; Hanson and Reshef 1997[25]) and tyrosine aminotransferase (TAT) (Schmid et al., 1987[49]; Ganss et al., 1994[20]). In addition, GCs also facilitate muscle protein breakdown and increase the supply of amino acids that serve as gluconeogenesis substrates (Lecker et al., 1999[33]). Using the strategy of subjecting diabetic (insulin dependent) rats to adrenalectomy and glucocorticoid treatment, Exton et al., were able to demonstrate that hepatic glucose output was a consequence of glucocorticoid-dependent gluconeogenesis and was found to be underlined by up-regulation of PEPCK (Exton et al., 1973[14]). Glucocorticoids have been reported to have distinct effects on key mediators involved in the insulin signaling pathway. Cortisone treatment, which caused increase in blood glucose and insulin, was reported to be associated with reduced phosphorylation of insulin receptor without changes in the total levels of it, as well as reduced levels of IRS1 in skeletal muscle (Giorgino et al., 1993[22]). Saad et al., observed that dexamethasone reduced stimulated insulin receptor phosphorylation status in livers of rats, along with reduced phosphorylation of IRS1 and PI3K activity associated with IRS1. Further, dexamethasone was also found to be associated with reduced IRS1-associated PI3K activity in muscle as well (Saad et al., 1993[48]). The glucose transporter, Glut4 is the main insulin-responsive transporter that mediates insulin-induced glucose uptake in skeletal muscle and adipose tissue. The insulin-responsiveness of glut4 is characterized by insulin-induced translocation of the transporter to the plasma membrane from intracellular locations. Short-term treatment of rats with dexamethasone resulted in decrease in insulin stimulated glucose uptake, which was found to be underlined by impairments in cell surface recruitment of glut4 to the plasma membrane (Weinstein et al., 1998[60]). 

With extensive research done, it is now apparent that excessive activation of glucocorticoid receptor plays a crucial role in the development of metabolic syndrome/T2D. Therefore glucocorticoid receptor antagonism may offer a viable approach for mitigating abnormalities associated with metabolic syndrome. This review intends to present an account of studies conducted with experimental animals as well as in human subjects that demonstrate efficacy of glucocorticoid antagonism in mitigating metabolic abnormalities typically associated with the metabolic syndrome.

The data on efficacy of GR antagonism in mitigating metabolic abnormalities is not limited to only preclinical studies. Human-subject based studies demonstrate that GR antagonism holds realistic promise in reducing the burden of metabolic abnormalities associated with excess glucocorticoids. It is important to recognize that much of our understanding of effect of mifepristone on glucocorticoid-related abnormalities has come from treatment of patients with Cushing's syndrome.

From studies conducted with various diabetic animal models, and studies conducted on Cushing's patients, it appears that GR antagonists have the potential to mitigate metabolic abnormalities associated with diabetes/metabolic syndrome. Despite its observed efficacy in alleviating anomalies associated with Cushing's syndrome, mifepristone has the disadvantage of lack of specificity (due to progesterone antagonism) and is associated with hypokalemia as a result of counter-regulatory activation of HPA axis-induced hypercortisolism (Castinetti et al., 2010[8]). While mifepristone is of great value in treating severe cases of Cushing's syndrome, selective GR antagonists that are devoid of propensity to cause activation of HPA axis are likely to be evaluated more for the possibility of therapeutic management of metabolic dysregulations associated with metabolic syndrome.

See also Table 1(Tab. 1) and 2(Tab. 2) (References in Table 1: Clapham and Turner, 1997[11]; Friedman et al., 1997[18]; Gettys et al., 1997[21]; Hashimoto et al., 2013[26]; Jacobson et al., 2005[28]; Kusunoki et al., 1995[30]; Langley and York, 1990[32]; Liang et al., 2005[34]; Okada et al.,1992[39]; Priyadarshini and Anuradha, 2017[44]; Takeshita et al., 2015[52]; Taylor et al., 2009[53]; Watts et al., 2005[57]; References in Table 2: Beaufrère et al., 1987[4]; Bertagna et al., 1986[5]; Chu et al., 2001[10]; Debono et al., 2013[12]; Fein et al., 2015[15]; Fleseriu et al., 2012[17]; Nieman et al., 1985[37]).

## Acknowledgements

Authors are thankful to Jain (Deemed to be University), Bangalore for the support.

## Conflict of interest

None.

## Figures and Tables

**Table 1:**
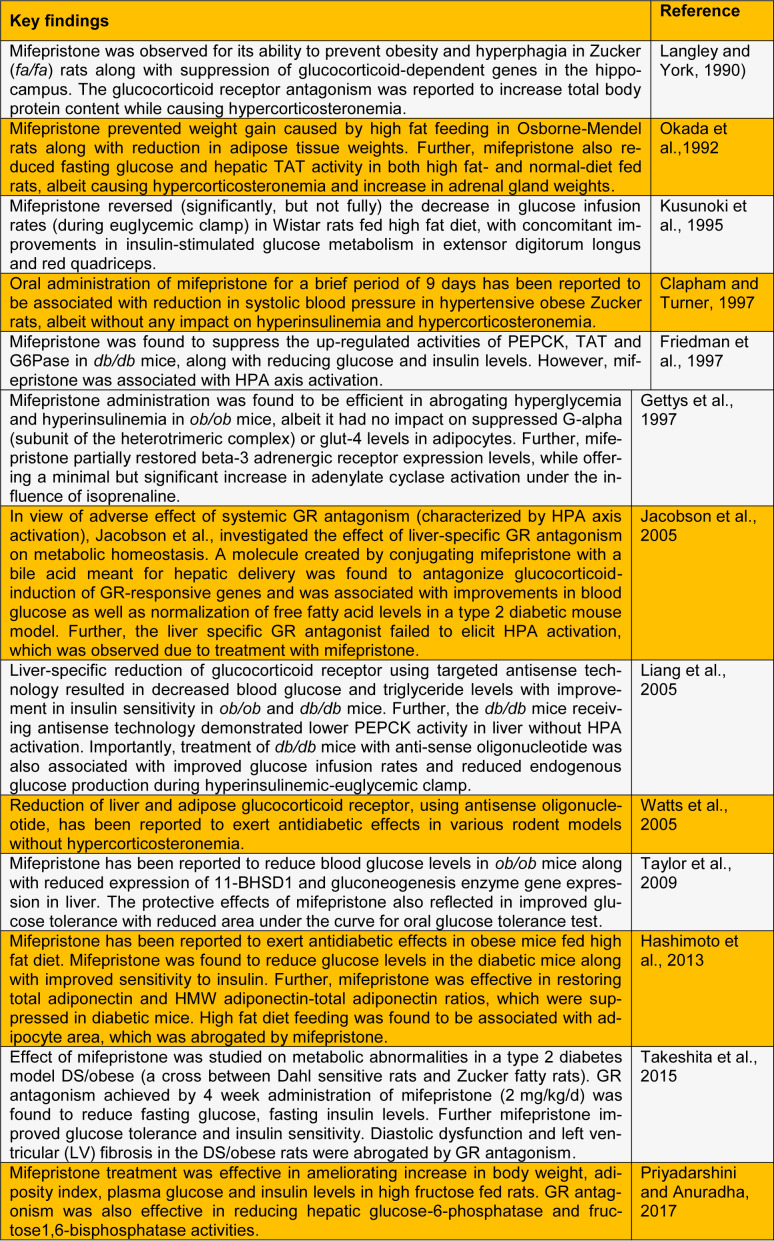
Summary of experimental reports on the effect of glucocorticoid antagonism on metabolic aberrations

**Table 2:**
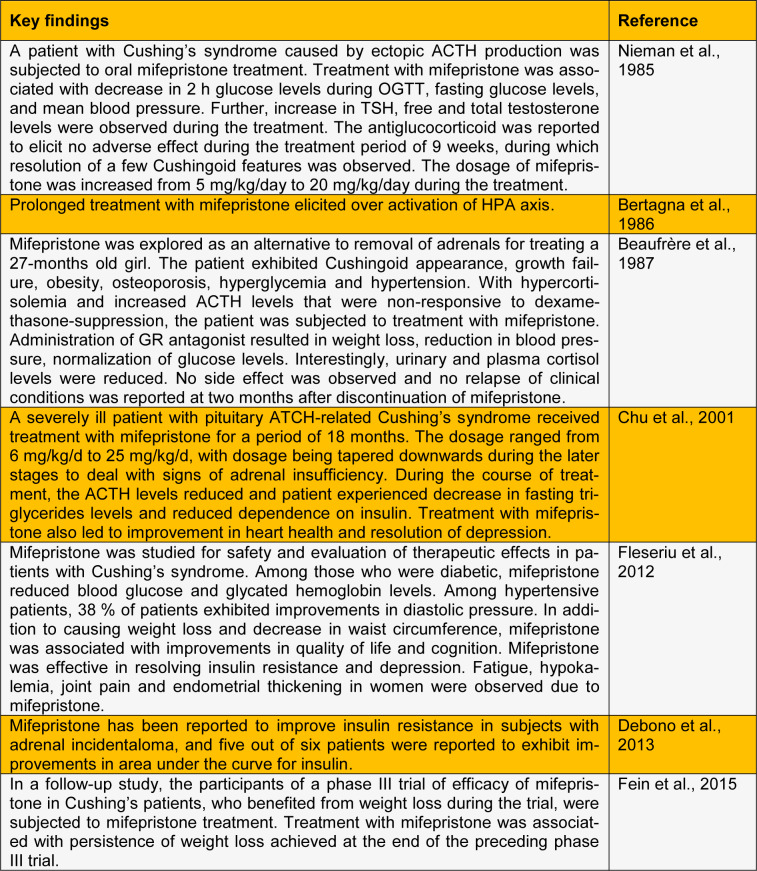
Human-subject based studies demonstrating effect of glucocorticoid antagonism on metabolic aberrations
